# Parliament and the Profession

**Published:** 1917-05-19

**Authors:** 


### PARLIAMENT AND THE PROFESSION.

### Army Nurses.

Mr. Macpherson assured Mr. Roberts that he would do his best to expedite the issue of the interim and final reports of the Supply of Nurses Committee, which has been before the Army Council for three and a half months.

### Doctors and Medical Service.

The nature of the replies given by the Under-Secretary for War to Mr. Watt indicated that there are a number of medical men in this country who are unemployed while awaiting their turns to go out or for relief.

### Omagh Guardians' Choice of a Medical Officer.

The Chief Secretary for Ireland said that he believed it was a fact that the Board of Guardians of the Omagh Union had elected to the position of medical officer of the district a Dr. M'Cartan, who is at present interned in England, and refused to appoint Dr. A. H. R. Duncan, a resident practitioner of Omagh, who has served two years with the Forces in France, where he was wounded in the eye.

### Shortage of Doctors.

Major Chapple asked the Secretary of State for War what number of medical officers are doing administration work in military hospitals; and whether two-thirds, if not all, of these could be replaced by non-medical officers in order to release men for professional duties. Mr. Macpherson stated that the number solely occupied with non-professional work is negligible, and that the administrative duties are so bound up with professional knowledge and experience that no saving in medical officers would result from increasing the number of non-medical officers now employed.

### Infected Blankets.

Mr. Macpherson stated that in order to prevent venereal and other disease being communicated to men sleeping in blankets previously used by infected persons, blankets are soaked in cresol solution before being washed, and he had no reason to suppose that these precautions are neglected.

### Endell Street Military Hospital.

Sir John Simon called the attention of the Under-Secretary of State for War to the terms of remuneration of the untrained nursing and administrative staff of the Military Hospital, Endell Street, as compared with other military hospitals. At Endell Street thirty-nine nurses are lodged outside the hospital at their own expense. Sir John asked if the staff could, under the recent orders, be given an increase in board and washing allowances, and have arranged for them adequate quarters or lodging allowances and pay as given to Voluntary Aid Detachments and probationers serving in other military hospitals. Mr. Macpherson promised to inquire into the matter.

